# The acceptability and feasibility of a pilot study examining the impact of a mobile technology-based intervention informed by behavioral economics to improve HIV knowledge and testing frequency among Latinx sexual minority men and transgender women

**DOI:** 10.1186/s12889-021-10335-5

**Published:** 2021-02-12

**Authors:** Sarah MacCarthy, Alexandra Mendoza-Graf, Zachary Wagner, Joanna L. Barreras, Alice Kim, Rebecca Giguere, Alex Carballo-Dieguez, Sebastian Linnemayr

**Affiliations:** 1 RAND Corporation, Behavioral and Policy Sciences, 1776 Main Street, Santa Monica, CA USA; 2 RAND Corporation, Pardee RAND Graduate School, 1776 Main Street, Santa Monica, CA USA; 3 RAND Corporation, Economics, Sociology, and Statistics, 1776 Main Street, Santa Monica, CA USA; 4 Bienestar Human Services, Inc., 5326 East Beverly Blvd, Los Angeles, CA 90022 USA; 5 School of Social Work, California State University, Long Beach, CA USA; 6 HIV Center for Clinical and Behavioral Studies, Division of Gender, Health and Sexuality, New York State Psychiatric Institute and Columbia University, 1051 Riverside Drive, Unit 15, New York, NY USA

**Keywords:** Behavioral economics, HIV prevention, Latinx, Sexual minority men, Transgender women, Feasibility, Acceptability

## Abstract

**Background:**

We developed a novel intervention that uses behavioral economics incentives and mobile-health text messages to increase HIV knowledge and testing frequency among Latinx sexual minority men and Latinx transgender women. Here we provide a theoretically-grounded assessment regarding the intervention’s acceptability and feasibility.

**Methods:**

We conducted 30-min exit interviews with a stratified sample of participants (*n* = 26 Latinx sexual minority men, 15 Latinx transgender women), supplemented with insights from study staff (*n* = 6). All interviews were recorded, transcribed, and translated for a content analysis using Dedoose. Cohen’s Kappa was 89.4% across coded excerpts. We evaluated acceptability based on how participants cognitively and emotionally reacted to the intervention and whether they considered it to be appropriate. We measured feasibility based on resource, scientific and process assessments (e.g., functionality of text messaging service, feedback on study recruitment procedures and surveys).

**Results:**

Regarding acceptability, most participants clearly understood the intervention as a program to receive information about HIV prevention methods through text messages. Participants who did not complete the intervention shared they did not fully understand what it entailed at their initial enrollment, and thought it was a one-time engagement and not an ongoing program. Though some participants with a higher level of education felt the information was simplistic, most appreciated moving beyond a narrow focus on HIV to include general information on sexually transmitted infections; drug use and impaired sexual decision-making; and differential risks associated with sexual positions and practices. Latinx transgender women in particular appreciated receiving information about Pre-Exposure Prophylaxis. While participants didn’t fully understand the exact chances of winning a prize in the quiz component, most enjoyed the quizzes and chance of winning a prize. Participants appreciated that the intervention required a minimal time investment. Participants shared that the intervention was generally culturally appropriate. Regarding feasibility, most participants reported the text message platform worked well though inactive participants consistently said technical difficulties led to their disengagement. Staff shared that clients had varying reactions to being approached while being tested for HIV, with some unwilling to enroll and others being very open and curious about the program. Both staff and participants relayed concerns regarding the length of the recruitment process and study surveys.

**Conclusions:**

Our theoretically-grounded assessment shows the intervention is both acceptable and feasible.

**Trial registration:**

The trial was registered on May 5, 2017 with the ClinicalTrials.gov registry [NCT03144336].

**Supplementary Information:**

The online version contains supplementary material available at 10.1186/s12889-021-10335-5.

## Background

HIV disproportionately affects Latinx sexual minority men (LSMM) and Latinx transgender women (LTGW), yet they are often unaware of their HIV status. The prevalence of HIV among the general U.S. population is less than 1%, [1] but HIV rates are markedly higher among LSMM and LTGW. In Los Angeles County, home to one of the country’s largest HIV epidemics [2] and site of the proposed study, the estimated HIV prevalence is 15% among LSMM [3] and 17% among LTGW [4]. Latinx adults are also less likely to have their infections diagnosed, [5] posing a critical public health problem as the undiagnosed population accounts for nearly 40% of new HIV infections [6]. A U.S. Centers for Disease Control report highlights the urgent need to increase HIV testing among individuals at higher risk for acquiring HIV such as LSMM and LTGW [7].

LSMM and LTGW face difficulties engaging with the formal health care system – often due to fear of engaging with perceived authorities such as providers - and mobile health (mHealth) approaches can help them access up-to-date HIV prevention information [8, 9]. MHealth technologies are a promising way to engage and remain in contact with communities facing a range of barriers in accessing health services. As the current COVID-19 pandemic shows, mHealth technologies can provide a crucial link to relay quickly evolving information. Recent studies [10–15] including our own [8, 16, 17] have shown how increasingly restrictive U.S. immigration policies [18] have heightened the unwillingness of many LSMM and LTGW to engage with formal systems, [19] further elevating the need to utilize mHealth platforms to maintain a critical line of communication. MHealth shows potential to improve HIV testing frequency among these groups, [20–25] but evidence of technology disengagement over time [26] makes it unclear how best to achieve this outcome [9, 27–30].

Our completed R34 study (MOTIVES – MObile Technology and IncentiVES), uses simple mHealth text messages combined with behavioral economics incentives, and produced extremely promising results [8, 31–34]. We partnered with Bienestar, Los Angeles County’s largest Latinx-serving HIV service organization. We randomized 166 participants into either the ‘*Information Only’* group (receiving weekly text messages with HIV prevention information) or the ‘*Information Plus’* group (who in addition could win small incentives conditional on responding to the quizzes). The quizzes included questions on the weekly text messages. Correctly answering questions improved the chance of winning a prize at the next testing visit from 1:10 if no correct answers to 1:5 if all correct. Our data show large effect sizes: 49% of participants in the ‘*Information Only’* group and 59% of the ‘*Information Plus’* group tested at Bienestar at least once in a year, compared to 35% in a matched control group (40 and 66% increases). Participants were also substantially more likely than controls to have at least two tests per year (78% increase in ‘*Information Only’* and 115% increase in *‘Information Plus’*) [34]. MOTIVES did not explicitly target Pre-Exposure Prophylaxis (PrEP - a daily pill that if taken consistently and correctly can reduce the risk of HIV from sex by about 99%) [35], but its uptake in the intervention groups more than doubled (*p* < 0.01).

In order to take the intervention to scale, a careful assessment of the intervention’s acceptability and feasibility are needed. Therefore, our goal is to provide a theoretically-grounded examination of intervention acceptability and feasibility to inform future scale-up and lessons learned for other mHealth interventions focused on sexual and gender minority populations such as LSMM and LTGW.

## Methods

Extensive details on the study design and quantitative data have been published elsewhere, [34] and here we provide a summary of key aspects and focus on the qualitative data that has not been previously described.

### Study aim, design and setting of the study

The goal of the study is to assess the acceptability and feasibility of the MOTIVES intervention. We designed the exit interviews to evaluate different domains of acceptability and feasibility based on theoretically-grounded frameworks (described in detail below).

The study was conducted at Bienestar Human Services, Inc., a community-based organization with multiple locations across Los Angeles County offering programs and services to primarily Latinx communities. Programs and services are available for HIV-positive people, individuals at risk for HIV, gay and bisexual men, lesbian women, transgender women, youth and people who use substances.

### Participant characteristics

The study sample included 41 study participants (*n* = 26 LSMM and 15 LTGW) and study staff (*n* = 6). Eligibility criteria for LSMM and LTGW participants were: being HIV-negative; self-identifying as a sexual minority man or transgender woman as well as Latinx; 18 years of age or older; owning or having regular mobile phone access; and fluency in English or Spanish. Staff participants included all providers enrolling LSMM and LTGW for the study.

### Procedures

**Fig. 1:**
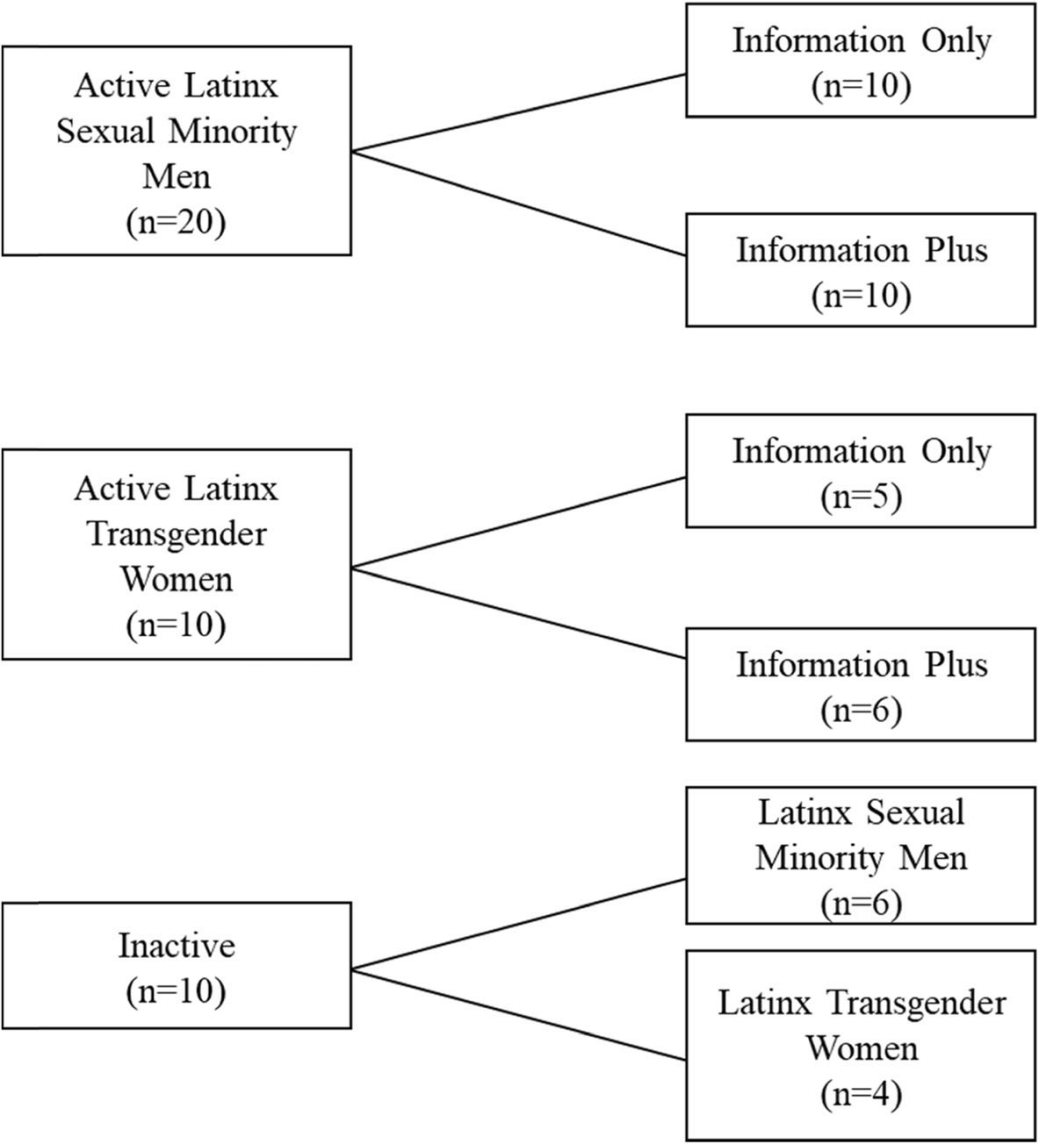
Exit Interview Sampling. Figure provides a detailed breakdown of the sampling frame for the exit interviews

The Institutional Review Board approved all study protocols and materials. Qualitative data was collected between April and June 2019. Participants provided written consent as part of their participation in the overall study, and for the phone interviews, all participants were read a copy of the informed consent document and provided verbal consent, as was approved by our Institutional Review Board. With respect to our stratified sampling approach, we were interested in speaking to people who were active participants as well as those who signed up for MOTIVES but never engaged in the program (inactive participants); those who were assigned to the ‘*Information Only’* component of the intervention and those who were in the ‘*Information Plus’* component. This gave us six groups to sample from (active LSMM in the ‘*Information Only’* component; active LSMM in the ‘*Information Plus’* component; active LTGW in the ‘*Information Only’* component; active LTGW in the ‘*Information Plus’* component; inactive LSMM; and inactive LTGW). We divided the study sample into these six groups, randomly selected participants within each of the groups to recruit for interviews and created a stratified sample that was proportional to the amount of total participants in each of the six groups. A breakdown of the sampling frame can be seen in Fig. 1 and descriptive statistics are included in Table 1.

**Table 1:** Socio-Demographic Characteristics of Latinx Sexual Minority Men (*n* = 26) and Latinx Transgender Women (*n* = 15)

	Latinx Sexual Minority Men (***n*** = 26)	Latinx Transgender Women (***n*** = 15)	Total (***n*** = 41)
*Background Characteristics*			
Mean age	37.36	37.35	37.36
At least a high school education	81%	47%	68%
Full-time employed	69%	53%	63%
Annual income less than $35,000	54%	73%	61%
*Documentation Status*			
U.S. citizen or permanent resident	67%	38%	57%
Deferred Action for Childhood Arrivals (DACA), asylum, and U visas for victims of violent crimes	13%	38%	22%
Undocumented	21%	23%	22%

All qualitative interviews were done by phone and conducted by a cisgender female, Latinx Assistant Policy Researcher from the RAND Corporation, who is fluent in both English and Spanish and is trained in qualitative data collection and analysis (AMG). Interviews were pilot tested several times, and various members of the research team provided feedback. All interviews were recorded, translated when necessary, and transcribed. Transcriptions and translations were carried out by an external transcription company. The translations were reviewed by the interviewer for quality assurance and were sent back for improvements when necessary, until high quality translations were obtained. The interviews lasted approximately 30 min, and participants were sent a $15 electronic gift card for participating in the qualitative interviews.

### Interview scripts to Assess Acceptability & Feasibility

We drew on theoretically-grounded frameworks in the peer-reviewed literature to discuss core components of acceptability [36] and feasibility [37]. We describe acceptability based on the framework provided by Sekhon and colleagues that assesses acceptability of an intervention based on cognitive and emotional responses to it. Tickle-Degnen suggests determining feasibility based on four types of assessments: management, resource, scientific, and process. Adequate management of the study and adequate resources to conduct it are requirements for NIH funding. Here we focus on the scientific and process assessments (e.g., reliability of our measurement tools, adherence to study recruitment) that determine the feasibility of large-scale implementation. Interview scripts for participants and study staff are included as Additional file 1.

### Qualitative data analysis

The approach to the data analysis was based on a directed content analysis, allowing for themes to emerge throughout an iterative coding process [38]. Two study investigators (SM and AMG) independently reviewed each transcript and then together developed a preliminary codebook, which was revised during joint coding of two subsequent interviews. The codebook included descriptions, inclusion/exclusion criteria, and example quotes. The final codebook included 10 higher-level, main themes and 16 sub-themes. To establish inter-rater reliability on the main themes, the coders jointly coded 10 transcripts and then randomly selected a set of 31 new transcript excerpts. Cohen’s Kappa was 89.4% across coded excerpts, indicating “good agreement” between reviewers [39]. After attainment of reliability, one coder coded the remainder of the client interviews. Because of the small number of staff interviews, illustrative quotes will not be included to maintain confidentiality. The two coders met weekly to review the work of one another and to identify emergent topics and discuss outliers. Finally, both coders reviewed all coded excerpts and collectively wrote a summary of results. Dedoose software (Version 8.3.21) was used to manage the qualitative data.

## Results

The below summarizes results regarding the intervention’s acceptability and feasibility. Illustrative quotes are included in Tables 2 and 3, respectively.

**Table 2:** Exemplary Quotes on Intervention Acceptability among Latinx Sexual Minority Men (*n* = 26) and Latinx Transgender Women (*n* = 15)

Acceptability Components	Themes	Exemplary Quotes from Latinx Sexual Minority Men	Exemplary Quotes from Latinx Transgender Women
Intervention Coherence	Understanding of the Intervention	“I would just say it’s like a course. It’s just like a few minutes a week just to gain an understanding of HIV.” (Active, Information Only)	“It’s a very professional study targeting...LGBT in general but definitely trans group and assisting us with prevention methods.” (Active, Information Only)
	Lack of Understanding of the Intervention	“They told me it was going to be a one-time thing.” (Inactive)	“I don’t remember receiving any information about what it was about.” (Inactive)
Affective Attitude	General Appreciation of the Information Received	“I loved how specific it was, and you guys didn’t just cater to gay sexuality either. You also talked about if you have sex with women, the vaginal fluids, and all that fun stuff – fun stuff, I guess. Yeah, you guys didn’t shy away from the topic. You approached it in a very adult manner, and I hope that people could grasp on that.” (Active, Information)	“I think that topics were very relevant and very helpful…For instance dealing with anal sex, dealing with preventative measures like PrEP, condom use.” (Active, Information Only)
Self-Efficacy	Appreciation for Quiz Component	“Everything was great. I liked it because it was like a game and I would get prizes and I would learn so everything seemed really good to me.” (Active, Information Plus)	“I liked it. The truth is I really liked it.” (Active, Information Plus)
	Lack of Understanding about Chances of Winning	“I really didn’t think I’d win anything. But I did get the gift card that was really, really nice.” (Active, Information Plus)	“Oh, well, the truth is I really don’t know how many people participate...If I knew how many there were, I would say, oh, I have a chance of winning.” (Active, Information Plus)
	Issues with Quiz Questions	“As for the questions, I did think that there were certain very specific questions where they were hard to answer. It could’ve been a yes or no. They weren’t worded properly.” (Active, Information Plus)	
	Use of Links	“Sometimes I [used the links] when I didn’t understand what the message was getting at.” (Active, Information Only)	“I would lie to you if I said that I always [used the links]. When I didn’t know the answer, I would click the link and learn a little more.” (Active, Information Plus)
Perceived Effectiveness	Increased Knowledge of HIV	“We think we know how you can actually protect yourself, and we think we know how you can help prevent it, but it’s not until you actually get exposed to the right information and you see cases. So, for me, I am grateful that I came across it simply because I feel more knowledgeable about it and better prepared.” (Active, Information Only)	“In general it helped me a lot to understand what this is, to understand that there are treatments out there, that perhaps the illness perhaps we might unfortunately might have it and it’s not the end of the world, there are treatments and things that help people have a normal life.” (Active, Information Plus)
	Continuation of Regular HIV Testing	“It helped me because I still do it. Even though I don’t get the messages now, it’s just like something that I learned. I do it now.” (Active, Information Only)	“You never know about the diseases and all that. And it’s very good because at least I get it almost every 3 months because you never know if you’re going to get the disease.” (Active, Information Only)
	Change in Sexual Behavior	“It actually improved because I was able to feel more comfortable about it and be able to have a better relationship. So, it definitely impacted me in a positive way with my partner.” (Active, Information Only)	“You never think about what you are going to do. You just do it, and if something you are going to do comes to your mind, then you are going to be careful…remembering everything you have learned.” (Active, Information Only)
	PrEP	“I had some knowledge, but really it was the program that gave me all this information.” (Active, Information Only)	“You learn that if you put yourself on PrEP, being in PrEP doesn’t mean that you’re not going to get HIV. You can get another disease, then everything has its consequences, and everything has its ups and downs.” (Active, Information Plus)
Burden	Minimal Effort	“[It took] like 2 minutes of my day. Well, I remember like getting every Thursday and Friday, but it was a quick simple thing.” (Active, Information Plus)	“[The effort] was minimal, and it was very convenient. Reading a text message takes less than 30 s.” (Active, Information Only)
Ethicality	Cultural Appropriateness of MOTIVES	*Appropriate for LSMM:* “The culture you have is very adequate, very adequate, they respect us, and I would also recommend it and I think that for other people, I mean, for other gay men, this is very good.” (Active, Information Plus)	*Appropriate for LTGW:* “It’s great that you have someone who worries about people like us...We live everyday just as it comes, and we don’t have any awareness about what is going on around us. We worry more about other things, about what a normal day is going to be like, perhaps, knowing that there are people that don’t accept us. So, we don’t worry about what’s inside or what’s more important, which is your health. And our physical health. So, it’s good that someone is doing something different for people like us.” (Active, Information Plus)
	Level of Comfort with Text Messages	“Even one of my friends took my phone and saw that I put it under Motives HIV for a contact name. And he said, what, what’s going on with you. I was like, no, it’s absolutely fine. I explained the research program. And he went, ‘oh, okay, well that’s interesting.’” (Active, Information Plus)	“Even if anybody would have read my text messages, which is not great, it wouldn’t have bothered me.” (Active, Information Only)
Opportunity Costs	Appreciation of Gift Cards	“I thought it was a great gift. Yeah. It’s definitely something worth taking.” (Active, Information Only)	“Well, I feel really good, I feel happy because it’s like a reward, you see, for our time.” (Active, Information Plus)
	No Need for the Gift Cards	“About the money I say I wouldn’t need to get it because I tell you, I like to go to that Bienestar program. I like to be informed about all this about HIV, the drugs, the treatments, all that. I even thought they were talking to me to find out more about PrEP and PEP.” (Active, Information Only)	“I think the people who participate are interested in the information they give. And I participated for that, for the information, not so much for other reasons.” (Inactive)

**Table 3:** Exemplary Quotes on Intervention Feasibility among Latinx Sexual Minority Men (*n* = 26) and Latinx Transgender Women (*n* = 15)

Feasibility Components	Themes	Exemplary Quotes from Latinx Sexual Minority Men	Exemplary Quotes from Latinx Transgender Women
Resource Assessment	Technical difficulties	“As far as I know, it was all going to be through text messages; the surveys were going to be via text messages, and they were going to send me texts. But honestly, I never received anything.” (Inactive)	“Oh yeah, they were supposed to send me text messages, that’s correct, but I haven’t received any. I did get them for a while and then I didn’t get anything.” (Inactive)
	Recommendations for technical issues	“Someone should have called me, but nobody did.” (Inactive)	“If the person would have helped me right there, and if I would have received a confirmation on my cell phone, but, as far as I remember, the person gave me a sheet of paper and said go to this website and follow the instructions and everything and that was it.” (Inactive)
Scientific Assessments	Intensity of Scientific Assessments	*Not a lot of time spent on surveys:* “The time was appropriate, although I wanted a little more. I understand you were very respectful of time.” (Active, Information Only)	*Not a lot of time spent on surveys:* “I didn’t feel it was a lot of time or anything. It was fine.” (Active, Information Only)
		*Easy to understand:* “They were like really user-friendly and they were to the point and they weren’t really like too like irrelevant...I feel like the questions they really applied to me, and I felt like it was a useful survey.” (Inactive)	*Easy to understand:* “I think [the questions] were easy, honestly, they were easy, you just have to use common sense.” (Active, Information Plus)

### Intervention acceptability

#### Intervention coherence – did participants understand MOTIVES?

Participants gave a range of descriptions of the intervention, but most said MOTIVES was a program where they received information about HIV prevention methods through text messages. However, when providing explanation as to why they did not continue with MOTIVES, some inactive participants shared they did not fully understand MOTIVES at their initial enrollment, and thought it was a one-time engagement rather than an ongoing program.

#### Affective attitude – how did participants feel about MOTIVES?

Most participants shared they had a good experience with MOTIVES. With regards to the structure of the intervention, participants said they found it useful to receive texts reminding them to get tested, and they liked getting texts on the same day of the week. Though some participants with a higher level of education felt the information was simplistic, most appreciated the content of MOTIVES because of the specificity and practicality of the information, as well as the variety of topics covered. Key topical areas that participants appreciated were: moving beyond a narrow focus on HIV to include general information on sexually transmitted infections; drug use and impaired sexual decision-making; and differential risks associated with sexual positions and practices. Additionally, many participants – and especially LTGW participants - appreciated receiving information about PrEP. Other topics participants thought should be integrated into the messages included individual-level concerns such as general mental health and more information on drug use, in addition to HIV-specific issues such as medical advancements and more on the side-effects of PrEP. Participants also said they wanted more information on structural-level issues such as knowing your rights with respect to accessing publicly available services and domestic violence. An example of an LTGW-specific concern included parenting for those who are transitioning.

#### Self-efficacy – were participants able to perform the MOTIVES activities?

Most seemed to enjoy the intervention and saw it as fun. With respect to the weekly text messages, most participants reported only having used the links a few times, primarily when they were less familiar with the topic covered in the text message. Participants generally participated in the quizzes, though some critiques of the quizzes were noted. For example, several said the quiz questions were poorly worded, and that they did not clearly understand the chances of winning.

#### Perceived effectiveness – did participants think MOTIVES was effective?

Participants shared positive changes in all three outcomes of interest for the intervention (i.e., knowledge about HIV, getting tested for HIV, and changing sexual behavior), as well as increased knowledge about PrEP. Most participants mentioned they learned new things about HIV, and even those who felt they already had adequate knowledge said they appreciated receiving updated information. In terms of getting tested for HIV, most participants shared intentions to continue getting tested every 3 months, though a few participants who were not sexually active shared they would likely only get tested every 6 months. With regard to changes in sexual behavior, participants mentioned feeling more confident about how to practice safer sex and feeling more comfortable asking their partner’s HIV status before having sex.

Despite the study not specifically targeting PrEP, we found a statistically significant increase in its uptake among MOTIVES participants [34]. Most participants said they had previously heard about it but learned a lot more about it through MOTIVES. Participants reporting that they were not currently on PrEP relayed individual-level barriers (e.g., not having many sex partners, concerned that it didn’t protect against other sexually transmitted infections, concerns about side effects and interaction with body affirming hormones for LTGW) as well as structural-level barriers (e.g., not having health insurance).

#### Burden – what was the perceived effort of participating in MOTIVES?

Participants liked that MOTIVES required a minimal investment of their time and said that it was both easy to participate in MOTIVES and that it did not take a lot of time out of their day. Some mentioned feeling like it took effort either because they spent more time thinking through the questions to improve the likelihood of answering correctly or because they were busy when the texts came in. Further, most were satisfied with the frequency and timing of the text messages, though some LTGW expressed wanting to receive messages more frequently, while a few LSMM expressed wanting to receive messages less frequently.

#### Ethicality – was the intervention a good fit with the participant’s value system?

Both LSMM and LTGW shared that MOTIVES was generally culturally appropriate. Many also said that the information received would likely be appropriate for a broader audience and should not be limited to just people in Latinx LGBT communities. Additionally, participants generally shared feeling comfortable with receiving information about HIV on their cell phones, with only one participant sharing feelings of discomfort.

#### Opportunity costs – were sufficient benefits given to engage participants in MOTIVES?

Most participants appreciated receiving gift cards for their participation in MOTIVES. Some felt the gift cards motivated them to stay engaged with the intervention, but many others said they would have likely participated in MOTIVES even without the gift cards. Many participants shared that the gift cards helped them buy essential items, like shampoo, eggs, milk, and condoms. Some reported difficulties with receiving their gift cards or with using their gift cards to pay for things. Suggestions for future incentives included having additional options for different kinds of gift cards and increasing the value of each gift card.

### Intervention feasibility

#### Management assessment

These components (e.g., investigators’ administrative capacity to manage the randomized controlled trial as well as the research investigators and staff capacities, expertise and availability for the planned research) were required to receive the grant. Other aspects (e.g., formats and structures of the trial) are described in detail elsewhere [34].

#### Resource assessment – technological capacity & software

Overall, most participants mentioned the use of text messages worked well. Of note, the main reason given for not continuing with the intervention related to technical difficulties. Almost all participants said that they believed they had signed up for the intervention and then simply never received subsequent text messages. Other examples of technical problems included participants being unsure of whether their quiz responses had been logged; receiving messages split up and out of order; and issues receiving their mobile gift cards. Several participants also noted not being signed up for their language of preference. Recommendations for technical issues included having a staff person on the spot send the message and have them confirm they received it; and having someone follow up to see whether or not they are receiving the messages as planned.

#### Resource assessment: institutional support – what is [the organization’s] willingness, motivation, and capacity to carry through with project-related tasks and support investigator time and effort?

Bienestar staff reported they felt it was important to work with LSMM and LTGW and that MOTIVES provided a better understanding of the study populations, uncovered some of the barriers they face in trying to remain HIV-negative, and provided critical information. The staff previously had varied levels of experience with research, with some having participated in research projects in the past and others having never been part of such a project. However, staff generally shared an interest in participating in future research activities, often because they wanted to make a difference and wanted to expand their skillset. Some did say they would be more motivated if additional compensation was made available, more recognition and involvement in other aspects of the research project (e.g., analysis, developing results), and had more flexibility in their workload to accommodate research activities. Further, most felt that it would not be difficult to gain supervisor approval to participate in future research activities, and they estimated they could spend between half an hour to 2 h per day working on research projects.

#### Process assessment – what is the recruitment process?

Staff shared that clients had varying reactions to being approached while being tested for HIV, with some unwilling to enroll and others being very open and curious about MOTIVES. There were a number of tactics that staff members found helpful for recruitment, including preparatory activities like having role playing scenarios and being able to ask questions among team members. However, there were also several challenges that staff members faced during recruitment. For instance, some felt that it was difficult to only be able to enroll walk-ins (in an effort to engage new rather than existing clients of Bienestar). Others had a difficult time enrolling people who were skeptical of the personal information being collected about them or of having information about HIV sent to their phones. Timing also seemed to be a challenge during recruitment, for both staff and prospective participants, with staff feeling like they had to juggle more priorities, and prospective participants feeling like the recruitment process took too long (between 30 and 40 min) when they had come in expecting a quick HIV test.

#### Scientific assessments – what is the level of safety and burdensomeness of the frequency, intensity, and duration of MOTIVES?

With respect to the surveys, most participants felt the survey was an appropriate length, with only a few sharing that they felt the surveys were too long – a sentiment that was echoed by some the study staff. Participants generally found the survey questions easy to understand; however, there were some who felt that the translations were done poorly, making some questions difficult to interpret. With respect to the midline and endline surveys, staff faced participant non-responsiveness or not keeping to appointments, as well as outdated participant contact information (e.g., either outdated emails, phone numbers, or contact information of a family or friend given at the start of the study). Staff found the following to be useful for getting participants to complete the surveys: explaining the purpose and importance of the research, stressing the gift card incentive, and being able to do some of the surveys over the phone rather than all in person (for participant convenience).

## Discussion

Our exit interviews provide a comprehensive assessment of the intervention’s acceptability and feasibility. Here we compare and contrast key issues raised with existing literature.

With respect to acceptability, most participants relayed high intervention coherence and said they understood MOTIVES, but some noted confusion in what the study entailed and how long it would last. Though we created a range of tools to ensure participants understood key aspects of the consenting (e.g., highlighting summary points after reading the full consent document) and study (e.g., visuals relaying the chances of winning), these findings underscore the need to identify engaging and helpful ways to ensure study participants understand core components of the research they are agreeing to participate in. The HIV Prevention Trials Network – a worldwide collaborative clinical trials network responsible for large randomized controlled trials globally – has called for improvements to these processes to ensure studies adequately address the informational needs of participants, [40] yet more concrete examples of tips and tools employed by researchers to achieve this understanding is needed.

The affective attitude (i.e. how people felt about MOTIVES), self-efficacy (i.e. whether participants were able to do the tasks) and perceived effectiveness relayed by participants was promising. Overall, participants appreciated the intervention and the fact that it required minimal time given their busy professional and personal lives. They thought the intervention was enjoyable (despite not fully understanding their exact chances of winning) and helped them improve key prevention behaviors. Our findings highlight the value of and need for such interventions in conjunction with others that address ongoing structural challenges [41].

Participants responded well to the tailoring of the intervention to the unique needs of LTGW, however participants reported confusion when asked if the intervention met their needs as a Latinx participant. A first-generation immigrant on our research team suggested this challenge may be due to the fact that, without significant interaction with other cultures (e.g., through higher education and other employment opportunities) it can be challenging to identify what is unique to your own culture without points of comparison. There is extensive literature on the importance of cultural tailoring [42] and even how to operationalize it [43, 44]. Beyond ensuring materials are accurately translated, that the right literacy levels are achieved, and that appropriate visual representations are included, our experience suggests that more nuanced domains of cultural competence remain hard to achieve. Perhaps most important is the meaningful engagement of the study population in all aspects of the study, from design through implementation. Further, integrating not just the feedback but ensuring the study population is represented in the staffing (both on the research team and study staff) is critical to ensuring diverse perspectives are not only considered but put into practice.

While the perceived opportunity costs of participation reflected here were minimal, we find somewhat contrasting results between participants’ preferences for gift cards. Specifically, in our formative interviews participants requested gift cards for fun activities such as going to the movies, and especially for LTGW, to buy make up at upscale stores [33]. However our exit interviews suggest that participants wanted more practical options to purchase food and other household items. One consistent point was that people wanted cash, but our community partner – like many others - have strict rules about the safety of storing cash at their offices for research purposes. Despite extensive investigation, our study team could not identify a credit card-like gift card that did not have fees attached to it – all existing options had prohibitively high fees that would have undermined the overall low-cost approach employed in the intervention and further limited the ability of future community organizations to include the incentive component. Going forward, providing a range of options that can be used to buy both essential and non-essential items may be ideal.

With respect to intervention feasibility, the use of technology was mentioned as both a strength and sometimes a weakness. While technology enabled a streamlining of many aspects of the intervention, it also introduced some challenges (e.g., changed phone numbers, inconsistent text messaging formats) and underscores the need for study staff to check in and confirm technological components of the intervention throughout the intervention, which in some ways undermines the essence of the light touch approach of mHealth. Other studies have also found mobile technology to be feasible for implementing health interventions to increase knowledge and improve health behaviors, [45–48] though some of them have also encountered difficulties during implementation, [49] suggesting the need for further exploration of the ways in which these interventions may be further improved.

Another overarching theme with respect to institutional and scientific assessments is the constant balance between programmatic and research goals. We designed the study with a focus on implementation science [50–54] to improve the integration of the research procedures into the existing workflow. Despite these efforts, study staff consistently raised concerns about the time constraints it placed on them. Collectively our team has over 20 years of HIV prevention research experience and to date this study included some of the most streamlined research instruments. Still, participant and study staff raised concerns about the time taken to complete research activities including consenting, surveys etc. [40] This emphasizes the need for ongoing discussion in order for study staff to better understand why the range of data are collected and how gathering more robust information allows for a more detailed understanding of the factors driving the intervention’s overall impact.

This study has both strengths and limitations. The strengths include theoretically-grounded assessments of acceptability and feasibility – two often talked about concepts in research that are however rarely rigorously assessed. Further, the study includes both participant and staff perspectives, helping to address issues raised from multiple vantage points. Finally, the structured sampling approach provided more balanced insights regarding the study’s strengths and weaknesses, rather than limited to only those who successfully completed the intervention and therefore were more likely biased in a positive direction. These strengths must be noted, however, in the context of limitations. First, exit interviews were all conducted at the end of the pilot study and would have garnered more recent recollections if conducted more immediately after dropping out or completing the study. Relatedly, the lapse in time made it particularly difficult to gather helpful information regarding participants who dropped out of the study shortly after it started (versus those who were recruited towards the end of the enrollment period and therefore conducted exit interviews more recently following their study completion). Second, due to the small size of our community partner, interviews with management-level staff would be impossible to appropriately de-identify; therefore, our discussion of institutional support is limited to study staff perspectives. Finally, exit interviews did not generate robust information on whether or not study procedures were followed beyond the recruitment process.

## Conclusions

Our theoretically-grounded assessment shows our intervention is both acceptable and feasible. While there are areas for improvement, the concerns raised are addressable. Further, our in-depth analysis provides a template regarding how best to comprehensively assess the diverse domains of acceptability and feasibility. Taken together, our research highlights the potential of this intervention and transparently discusses the ways in which its acceptability and feasibility can be improved going forward.

## Supplementary Information

**Additional file 1.**

## Data Availability

Data underlying the study cannot be shared publicly or on request due to ethical restrictions imposed by the authors’ ethics committee, the Certificate of Confidentiality received from the NIH, and the consent form signed by participants. Any questions about this can be directed to Sarah MacCarthy through the following modes of communication: Phone: 310–393-0411, ext. 6743. Fax: 310–393-4818. E-mail: sarahm@rand.org

## Abbreviations

LTGW: Latinx transgender women
LSMM: Latinx sexual minority men
PrEP: Pre-Exposure Prophylaxis
MOTIVES: MObile Technology and IncentiVES
mHealth: Mobile health
