# Supplementary material for: The acceptability and feasibility of a pilot study examining the impact of a mobile technology-based intervention informed by behavioral economics to improve HIV knowledge and testing frequency among Latinx sexual minority men and transgender women
Source: BMC Public Health. 2021 Feb 12;21:341. doi: 10.1186/s12889-021-10335-5 (PMC7880516; doi:10.1186/s12889-021-10335-5)
Supplement: Supplementary file 1 — Additional file 1. [file 12889_2021_10335_MOESM1_ESM.zip › MOTIVES exit interview guide_ParticipantsR4.docx]

**General:** *First we’d like to know your thoughts about MOTIVES.*

**In general, what did you think of MOTIVES?** (If good or bad, ask about what specific things were bad or good and why)

- How woud you describe MOTIVES to someone else?
- How much time and effort did it take for you to participate in MOTIVES? (Was it difficult or easy to participate? Did it take a lot of time or a little bit of time? What took a lot of time? What didn’t take much time?)
- Good or bad experiences? Why? (For good, what would be some of the highlights? For bad, what would be some of the things you didn’t like and why?
- Most\Least useful parts? Why?
- Recommend to other (use one: Latino gay men or Latina trans women)? Why or why not? (If yes, I can imagine why, but tell me more; if no, also ask them to tell a bit more about why)

**Intervention Content**: *Next we’d like to know what you think of the different parts of MOTIVES.*

**What did you think of the information included in the texts?**

- Other issues or topics to include?
- Was there anything in the text messages that made you feel uncomfortable?
- Did you use the links to website information? If so were the links useful? If not, why not?
- Did you have any issues or concerns with HIV information being sent to your phone?

**Text Message: What did you think about the number of texts sent? Was it too much, too little, or just right? Why?**

**Incentives:** **What did you think about the incentives?**

- What did you think about the incentives for taking the first and last surveys? What about the incentive you got for doing the survey in the middle?
- (For quiz groups only)
  - What did you think about the amount you won as a result of the getting the quiz questions right? Did it encourage you to do the quiz or would you have done it anyway?
  - What did you think about the frequency, or how often the quizzes were sent to you?
  - Did you understand your chances of winning?

**Cultural Sensitivity:** *We would like to know what you think about the program specifically for use with (Choose one: other Latino gay or bisexual men / Latina trans women).*

- How much did you feel that MOTIVES was culturally sensitive, meaning they helped to meet the needs of (choose one: Latino gay men or Latina trans women)?
- What parts of MOTIVES were? What parts of MOTIVES weren’t?
- How could MOTIVES be more useful for (choose one: Latino gay or bisexual men or Latina trans women)?

**In what ways do you think MOTIVES can be improved or changed?**

Feasibility of the Survey Content and Length: *We’d like to know what you thought about the surveys we did during the study.*

- How did you feel about the first and last survey? Were they too long, too many questions, difficult or easy to do?
- How did you feel about the survey in the middle? Was it too long, too many questions, difficulty or easy to do?

Perceived Effects: *We’d like to know how MOTIVES may impact you now that it is over.*

- How much do you think MOTIVES changed how much you know about HIV?
- How much do you think MOTIVES will help you in getting tested for HIV every 3 months going forward?
  - How did you feel about the time it took to get tested?
- How much do you think MOTIVES changed your sexual behavior?

*Thank you very much for your feedback about MOTIVES. We will use it to improve the program for the next phase of the stuy.*
