# Supplementary material for: The acceptability and feasibility of a pilot study examining the impact of a mobile technology-based intervention informed by behavioral economics to improve HIV knowledge and testing frequency among Latinx sexual minority men and transgender women
Source: BMC Public Health. 2021 Feb 12;21:341. doi: 10.1186/s12889-021-10335-5 (PMC7880516; doi:10.1186/s12889-021-10335-5)
Supplement: Supplementary file 1 — Additional file 1. [file 12889_2021_10335_MOESM1_ESM.zip › MOTIVES exit interview guide_ProvidersR4.docx]

1. For how long have you worked in Bienestar? What led you to take this job?
2. What are your main responsibilities? What additional responsibilities do you have?
   - I notice that you did/did not mention being directly involved in research. Please tell me more about that.
   - From your point of view, what is the importance/lack of importance of doing research with the type of clients that you see?
3. The focus of our study was on trying to get uninfected people to check their HIV status with regularity, hopefully every three months. In your view, how much of a priority should be given to this issue?
   - Are there other issues that you think need research and that in your view are more important than this one?
   - What kind of issues puzzle or confuse you about your clients? Please describe some things that are hard for you to understand about your clients (e.g. repeated risk taking despite receiving counseling).
4. To what extent have you worked with researchers before?
   - What was that experience like?
   - What role, if any, would you like to have in research more generally?
5. If we decided to build on the recent experience and design a new study, please tell us:
   - To what extent would you be interested in participating in the initial development process, that is, deciding on what the study would look like?
   - How much time per week or per month do you think you could dedicate to be part of the planing process?
   - How easy or difficult would it be to get clearance from your supervisors to dedicate time to a project like this?
   - What would make you enthusiastic about participating in similar studies in the future? (e.g., extra provider incentives, a chance to win a lottery prize themselves, more training opportunities, a certificate of appreciation for their work on a research study)
6. How would you describe your experience with the MOTIVES study in general?
   - - Good or bad experiences? Why?
     - Most\Least useful parts? Why?
     - Recommend to other Latino MSM / Latina transgender women? Why or why not?
7. We’d like to know about the recruitment process:

- How did participants respond when you approached them about the study?
- What helped you during the recruitment process?
- What was challenging during the recruitment process?
- What would you do differently in the future?

1. We’d like to know about the baseline survey:

- What helped you to get clients to complete the baseline survey?
- What made it difficult to get clients to complete the baseline survey?
- What would you do differently in the future?

1. We’d like to know about the final survey:

- What helped you to get clients to complete the final survey?
- What made it difficult to get clients to complete the final survey?
- What would you do differently in the future?

1. We have some final questions about the surveys content and length:

- How much did you need to help participants to complete the surveys?
- Which questions or sections caused confusion?
- How was the survey length?
- How well did the surveys fit in with clinic flow and what could it be improved?

1. We’d like to know how MOTIVES may impact your clients now that it is over.

- How much do you think MOTIVES changed your clients’ knowledge and understanding about HIV?
- How much do you think MOTIVES will help in getting your clients tested for HIV every 3 months?
- How much do you think MOTIVES changed the sexual behavior of your clients?

1. Looking back at the way you saw MOTIVES being implemented, what advice can you give us on how to do things better?
2. Your time is valuable, so I don’t want to keep you any longer. What is the take home message you have for us?

*Thank you very much for your feedback about the program. We will use it to improve the program for the next phase of the study.*
